# Bioreactor Systems for Plant Cell Cultivation at the Institute of Plant Physiology of the Russian Academy of Sciences: 50 Years of Technology Evolution from Laboratory to Industrial Implications

**DOI:** 10.3390/plants13030430

**Published:** 2024-02-01

**Authors:** Maria Titova, Elena Popova, Alexander Nosov

**Affiliations:** 1 K.A. Timiryazev Institute of Plant Physiology, Russian Academy of Sciences, 127276 Moscow, Russia; elena_aygol@hotmail.com (E.P.); al_nosov@mail.ru (A.N.); 2 Department of Biology, M.V. Lomonosov Moscow State University, 119234 Moscow, Russia

**Keywords:** plant cell culture, cell suspension, bioreactors, biotechnology, periodic cultivation, continuous cultivation, phytochemicals, plant secondary metabolites

## Abstract

The cultivation of plant cells in large-scale bioreactor systems has long been considered a promising alternative for the overexploitation of wild plants as a source of bioactive phytochemicals. This idea, however, faced multiple constraints upon realization, resulting in very few examples of technologically feasible and economically effective biotechnological companies. The bioreactor cultivation of plant cells is challenging. Even well-growing and highly biosynthetically potent cell lines require a thorough optimization of cultivation parameters when upscaling the cultivation process from laboratory to industrial volumes. The optimization includes, but is not limited to, the bioreactor’s shape and design, cultivation regime (batch, fed-batch, continuous, semi-continuous), aeration, homogenization, anti-foaming measures, etc., while maintaining a high biomass and metabolite production. Based on the literature data and our experience, the cell cultures often demonstrate cell line- or species-specific responses to parameter changes, with the dissolved oxygen concentration (pO_2_) and shear stress caused by stirring being frequent growth-limiting factors. The mass transfer coefficient also plays a vital role in upscaling the cultivation process from smaller to larger volumes. The Experimental Biotechnological Facility at the K.A. Timiryazev Institute of Plant Physiology has operated since the 1970s and currently hosts a cascade of bioreactors from the laboratory (20 L) to the pilot (75 L) and a semi-industrial volume (630 L) adapted for the cultivation of plant cells. In this review, we discuss the most appealing cases of the cell cultivation process’s adaptation to bioreactor conditions featuring the cell cultures of medicinal plants *Dioscorea deltoidea* Wall. ex Griseb., *Taxus wallichiana* Zucc., *Stephania glabra* (Roxb.) Miers, *Panax japonicus* (T. Nees) C.A.Mey., *Polyscias filicifolia* (C. Moore ex E. Fourn.) L.H. Bailey, and *P. fruticosa* L. Harms. The results of cell cultivation in bioreactors of different types and designs using various cultivation regimes are covered and compared with the literature data. We also discuss the role of the critical factors affecting cell behavior in bioreactors with large volumes.

## 1. Introduction

Over 100,000 plant-produced secondary metabolites have been identified to date, and the discovery of new compounds continues on a daily basis. Plant secondary metabolites possess extremely diverse chemical structures. The alkaloids, isoprenoids (terpenoids), and phenolic compounds are the most well-studied metabolite classes so far, each of them being subdivided into numerous subgroups composed of thousands of chemicals. Other bioactive molecules belong to plant amines, non-protein amino acids, cyanogenic glycosides, glucosinolates, polyacetylenes, betalaines, alkylamides, thiophenes, etc. [1,2,3,4].

Many of the identified plant secondary metabolites are economically important products and are widely used in the pharmacological, cosmetic, and food industries, veterinary medicine and agriculture. Anticancer drugs, adaptogens, immunostimulants, and antimicrobials remain in the greatest demand [1,5,6,7,8,9,10]. The development of new synthetic drugs may cost about 100 million USD and take about 10 years; hence, the practical interest in plants as natural raw materials for drug production is economically justified [11,12,13].

The ever-increasing demand for bioactive compounds of plant origin leads to the overexploitation of wild plant diversity and, as a consequence, to the search for alternative renewable sources of valuable secondary metabolites. Plantations may partially solve the problem but have other issues such as significant costs, the use of herbicides and insecticides, land occupation, certain climatic conditions requirements, etc. Moreover, both the composition and the number of secondary metabolites produced by plantation plants are subject to change depending on the plant’s age and growth environments. Plant cell cultures provide fundamentally different opportunities for the production of plant bioactive compounds [4,14,15,16,17].

Cell biotechnology for the production of bioactive phytochemicals has numerous advantages over traditional plant raw materials as highlighted in earlier reviews [15,16,18,19,20]. The main advantages are independence from seasonal, climatic, and soil conditions, eco-friendly production process, a variety of strategies available for maximizing the yield of cell biomass and the compounds of interest, unlimited application to plant species with rare/endangered/at-risk status, and the use of standard equipment designed for microbiological productions including bioreactors, post-fermentation systems, etc., with only minor modifications. Recent advances in bioengineering and in vitro selection technologies may prompt the development of superior cell lines with intensive growth and the production of the desired metabolites at similar or higher levels compared to wild plants [21,22,23].

Recent advances in in vitro cultivation methods underpinned the development of both practical and fundamental aspects of plant cell and tissue culture [13,16,24]. In addition to biotechnological applications, plant cell cultures have been used to investigate the biosynthetic pathways and regulatory processes in plants and successfully utilized as natural systems for biotransformation, both at the final and intermediate production stages [25,26].

Numerous studies conducted since the 1940s have demonstrated that suspension cultures of plant cells are capable of synthesizing the entire range of secondary metabolites, often in amounts exceeding their concentration in plants [13,15,19,23,27,28,29,30]. Furthermore, plant cell cultures possess a high potential to synthesize phytochemicals that are found in a minority in donor plants [23]. Suspension cultures of plant cells can also be used to produce therapeutic proteins, including monoclonal antibodies, human serum albumin, human hemoglobin, interferon, immunostimulatory allergenic proteins, and others [13,25,31,32,33].

The list of plant cell cultures that have been tested for biotechnological application is quite wide and is constantly updated with new species. The most known and described in the literature are cell cultures of *Panax* spp. producing ginsenosides, *Taxus* spp. synthesizing paclitaxel, *Dioscorea* spp. producing steroidal glycosides, *Coleus blumei* Benth. producing rosmarinic acid, *Aralia cordata* Thunb. producing anthocyanins, *Lithospermum erythrorhizon* var. erythrorhizon Siebold and Zucc. producing shikonin derivatives, etc. [15,19,22,28,34,35,36,37]. Until the 2000s, plant cell-based biotechnologies have been mainly considered most relevant for the products that are unprofitable or unfeasible to manufacture using traditional methods of wild plant collections or plantation cultivation, for example, for bioactive metabolites produced by rare, endemic, or slowly growing plants [38,39]. To date, only a few effective biotechnological productions based on the large-scale cultivation of plant cell suspensions have been described [17,32]. The reasons for the limited industrial application of plant cell cultures are the high cost and the complexity of the hardware design, resulting in high production costs and the uncompetitively high prices of the final product as well as difficulties in developing productive cell strains [17,40,41].

**Figure 1:**
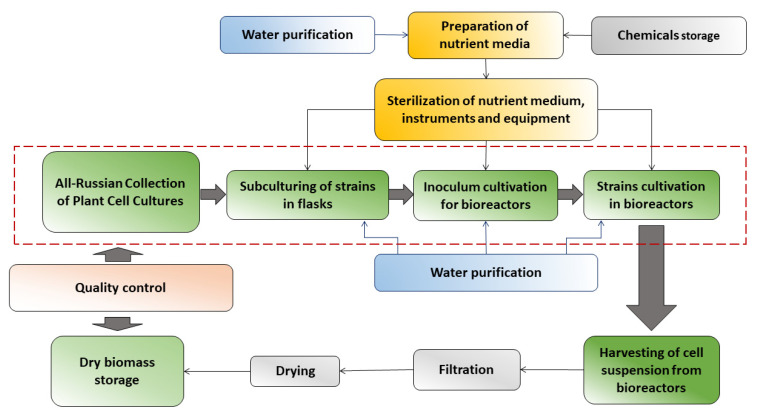
A simplified process flow chart of the Experimental Biotechnological Facility of the IPPRAS.

**Figure 2:**
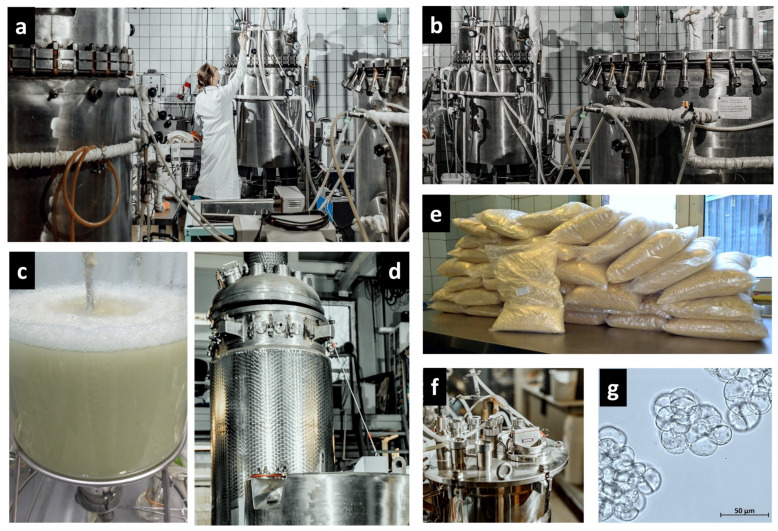
Experimental Biotechnological Facility of the IPPRAS: (**a**–**d**,**f**) bioreactors of different volumes for cultivation of plant cell suspensions; (**e**) cell biomass harvested from 630 L bioreactors, dried and packed for shipment; (**g**) example of suspension cells under microscope: cells of *Panax japonicus* strain 62 adapted for cultivation in industrial bioreactors.

The K.A. Timiryazev Institute of Plant Physiology of the Russian Academy of Sciences (IPPRAS) is a research institute that performed pioneer studies in different aspects of plant cell cultures [42,43,44,45]. The institute hosts the Experimental Biotechnological Facility, a large department with unique bioreactor systems specifically designed for the cultivation of plant suspension cell cultures from the laboratory (2–20 L) to the pilot (75 L) and the industrial (630 L) scale (Figure 1 and Figure 2). The facility serves both scientific and commercial applications, with the main goals of obtaining and selecting cell lines with enhanced production of bioactive metabolites, optimization of the cell cultivation conditions by adapting nutrient media and cultivation regimes, the upscaling of cultivation to industrial volumes, and the design and modification of equipment for cell cultivation. The research team conducts comprehensive studies aiming at developing the large-scale bioreactor cultivation of cell culture producers of biologically active compounds, taking into account the productivity and individual physiological characteristics of cell strains and the technological characteristics of the equipment used.

Here, we provide a historical perspective and a comprehensive review of our experience gained through several decades of plant cell cultivation using bioreactor systems of different types, volumes, designs, and operation regimes. Most studies were performed using cell culture strains from the All-Russian Collection of Plant Cell Cultures of IPPRAS featured in the previous review [22]. Each section gives a brief introduction to the different aspects of bioreactor cultivation and discusses their implications for plant cell cultures based on the literature and our experience.

## 2. Bioreactor Types for Plant Cell Cultivation and Their Specifics

The bioreactor system for plant cell cultivation had evolved from microbial production and is mainly based on the same principles but accomplished its own specifics. From the technological perspective, the process of living organisms’ cultivation in a bioreactor involves “in” and “out” flows: inoculum, air or gas mixtures, nutrient components, defoamers, etc. are supplied into bioreactors, constantly or periodically, while heat, exhaust air, culture medium, and cell biomass are removed from the system. The process is controlled by measuring the main physical and chemical parameters and their stabilization at the optimum level for maximizing the yield of the desired product (biomass or compound of interest). In the process of cultivation, a complex mixture is formed composed of cells and cell aggregates, extracellular metabolites, and residual concentrations of the initial substrate, while target products are usually found in small concentrations and may be easily destroyed [46,47,48,49,50]. These general principles are followed in the bioreactor cultivation of plant cells with certain specifics of regime optimization and bioreactor design and construction [15,51,52]. Most authors acknowledged that the major differences between microbial and plant cell production systems are due to the following specifics [15,19,20,51,53]:

- The large size and vacuoles make plant cells particularly sensitive to physical and mechanical stresses;
- The specifically high requirements for maintaining aseptic conditions during long cultivation due to the relatively low growth rate and long cultivation cycle of plant cell cultures compared to microbial and animal cell cultures;
- The high requirements of uniform mixing due to the high sedimentation rate of cell aggregates and the increasing viscosity of cell suspensions at the high concentrations of cell biomass;
- The intensive foaming and adhesion of cell biomass to the walls of a bioreactor;
- The complex mechanisms of regulating the cell growth and biosynthesis of target metabolites.

To meet these specifics, bioreactors for plant cell cultivation should be both structurally and operationally complex. The main engineering challenges are to maintain a high intensity of mass and energy exchange between the cells and culture medium, to minimize cell damage during mixing, to aseptically monitor technological parameters, in particular, the concentrations of cell biomass and target metabolites, and to minimize the cost of the process [15,52,54].

### 2.1. Bioreactor Classification Based on Their Design

Different types of bioreactors varying in design, operating regime, and size, from several liters to several thousands of tons, have been tested for plant cell cultivation. The large spectrum of cell cultures led to a huge variety of engineering solutions based on cell strain characteristics, medium used, production scale, specifics of the product isolation, etc. [15,52,54,55,56]. Several attempts have been made to classify bioreactors for plant cell cultivation. In the literature, bioreactors are most often grouped based on their constructions [20,52,54,55,56] into the following types:

- Bioreactors where mixing is performed by compressed air supply;
- Bioreactors with mechanical stirring;
- Wave bioreactors.

These types of bioreactors have been reviewed in detail [15,55]. Here, we provide only a brief description of their main characteristics that are important for further reading.

***Bioreactors with air mixing.*** In this type of bioreactor, the aeration and mixing of the cell suspension are performed by compressed air. The most well-known are the bubble type, airlift bioreactors that are usually shaped as a vertical tank equipped with a gas distribution device or spargers installed at the bottom. Such a construction is relatively simple, with no rubbing or moving parts, but highly functional and reliable [57,58]. This type of bioreactor was used in the first experiments on the scaling-up cultivation of plant cell suspension cultures [59], followed by decades of effective exploitation. Bubble-type bioreactors were employed for experimental cultivation of *Taxus cuspidata* Siebold. and Zucc. and *Eurycoma longifolia* Jack cells [60,61]. Several cases of commercial cultivation based on suspension cell cultures of *Panax ginseng* C.A. Mey., *Digitalis lanata* Ehrh., *Lithospermum erythrorhizon*, *Taxus baccata* Thunb., and *Taxus wallichiana* Zucc. in airlift bioreactors were also reported [36,40,62,63,64]. However, a number of studies demonstrated that bubble-type and airlift bioreactors have relatively low mass transfer characteristics, and they are, therefore, not recommended for cell suspensions with a high viscosity or high final cell biomass concentration [57,58].

***Bioreactors with mechanical stirring***. In these bioreactors, aeration is performed by compressed air, while a mechanical stirrer is used for mixing. Usually, these bioreactors are made as cylindrical vessels equipped with mechanical stirring devices and a sparger, which is normally installed under the bottom tier of the stirring device. The oxygen mass transfer coefficient values in these bioreactors may vary within a very wide range [55,58,65,66]. According to the literature, bioreactors with mechanical stirring are most widely used for upscaling the cultivation process from the laboratory to industrial volumes [67,68]. One of the first large-scale commercial bioreactor systems of this type was developed by the Diversa (later Phyton Biotech) company (Germany) and employed a cascade of five mechanically stirred bioreactors (75, 750, 7500, 15,000, and 75,000 L). This system was used for the cultivation of *Echinacea purpurea* (L.) Moench, *Rauvolfia serpentina* Benth. ex Kurz cell suspensions [69], followed by *Taxus chinensis* Roxb. for the production of paclitaxel [17]. Other examples of the successful cultivation of plant cells in bioreactors with mechanical stirring include *Panax* spp., *Catharanthus roseus* (L.) G. Don, *Podophyllum hexandrum* Royle, *Azadirachta indica* A. Juss., and some others [70,71,72,73,74].

***Wave bioreactors*** are rather complex in design, consume a substantial amount of energy, and are rarely used for the large-scale commercial cultivation of plant cells. They are characterized by wave-induced motion, where the mass and energy transfer are manually adjusted via the rocking angle, agitation rate, medium level, and culture vessel geometry. The disadvantages of these bioreactors are high energy losses during liquid agitation, engineering difficulties due to the lack of reliable methods for the calculation of optimum parameters, and the relatively high cost of additional equipment [55,75,76].

The given examples cover the most widely used industrial and laboratory types of bioreactors. However, new experimental bioreactors are constantly being developed, which are quite difficult to classify and do not fall into the usual categories [76,77,78].

### 2.2. Bioreactors of the Experimental Biotechnological Facility of the IPPRAS

The Experimental Biotechnological Facility of the IPPRAS has been operating since the 1970s and was the first Russian facility focused specifically on the bioreactor cultivation of plant cells. During its operation history, bioreactors of different types and volumes were tested and adapted for the cultivation of plant cell suspensions (Table 1). The very first experiments were performed in 1972–1979 using laboratory glass barbotage V-shape bioreactors (no. 1 in Table 1, total volume 1.5–3.0 L) and laboratory bioreactors MF-107 with a modified mechanical stirrer (no. 5 in Table 1, total volume 7.0 L). These bioreactors provided satisfactory conditions for both growth and the triterpene glycoside biosynthesis of the suspension cell cultures of *Dioscorea deltoidea* Wall. ex Griseb. in a series of physiological and biochemical studies [42]. The same systems were used in later experiments for culturing other strains of *Dioscorea deltoidea* [45,79,80,81,82], as well as for the suspension cell cultures of *Polyscias filicifolia* (C. Moore ex E. Fourn.) L.H. Bailey [83], *Panax ginseng* [80], and *Taxus baccata* [84]. Mechanically stirred laboratory bioreactors Fermus-apparatus and AK-210 (no. 3 и 4 in Table 1) were also actively operated in the 1990s, in particular for developing and optimizing cultivation regimes for different strains of *Dioscorea deltoidea* cell suspensions [85]. However, these bioreactors had various design flaws, and their current use is restricted to experimental purposes with a very limited number of suspension cell strains (Table 1).

During the past 20 years, the Biotechnological Facility of the IPPRAS primarily used laboratory glass bubble-type 10 L and 20 L bioreactors (No. 2, Table 1) or stainless steel 75 L and 630 L tank bioreactors (No. 6 and 7, Table 1) for plant cell suspension cultivation. Our studies confirmed that these bioreactors were the most favorable for the cultivation of undifferentiated plant cells and had fewer disadvantages compared to bioreactors of other types [21,80,87,88,89,90].

## 3. Cultivation Regimes

### 3.1. Cultivation Regimes Suitable for Plant Cell Cultures

The choice of bioreactor design and cultivation regime for maximizing the yield of biomass and/or a product of interest is mainly determined by the growth and biosynthetic characteristics of individual cell culture strains [15,32]. The most common cultivation regimes and their implications for culturing cells of different plant species are discussed below.

**The batch, or periodic, cultivation** is a type of “closed” cultivation when the system undergoes dynamic changes that might be difficult to control. The concentrations of cells and nutrient medium components, products of cell metabolism, and other factors are constantly changing in the course of the cultivation process following the development of the cell population inside the bioreactor [32,91]. The main advantages of batch systems are the following:

- A reduced risk of contamination and cell mutations due to the relatively short cultivation cycle compared to other regimes;
- The high degree of substrate utilization;
- The relatively low cost (compared to the cost of continuous cultivation).

Because of its simplicity and universality, this method has been widely used in plant cell cultivation experiments and industrial-scale productions, for example, for the cell cultivation of *Azadirachta indica* (azadirachtin), *Catharanthus roseus* (ajmalicine, catharanthine, serpentine alkaloids), *Panax notoginseng* (Burkill) F.H. Chen (ginsenosides), and *Taxus cuspidata* (taxane production) [29,60,92,93,94]. Hibino et al. [95] reported the cultivation of the suspension culture of *Panax ginseng* cells in 20,000 and 25,000 L bioreactors with mechanical stirring (periodic regime) with biomass productivity reaching 20 g dry weight (DW) (L·day)^−1^ in four weeks.

However, some authors noted the reduction in the cell biomass and secondary metabolite accumulation during cell cultivation as a result of batch cultivation compared to other regimes. This may be due to a gradual accumulation of the inhibitory products of cell metabolism in the medium and depletion of substrate during cultivation. In addition, the efficiency of the process is corrupted by the cultivation pauses for equipment preparation including cleaning, refilling, and sterilization of the bioreactor, as well as for preparing the required amount of cell culture inoculum for every new cultivation cycle. Upon upscaling to industrial volumes, there is an additional risk of contamination from inoculating bioreactors with substantial volumes of cell suspension [15,32,91].

**The continuous regime** for plant cell cultivation are mainly open systems; they can be organized according to the principle of complete mixing [91,96]. During continuous cultivation, fresh medium is continuously fed into the system at a constant rate under mixing, whereas the total volume of the cell suspension is kept stable by continuously removing a portion of the suspension culture at the same rate. A continuous regime creates uniform stationary conditions in the whole volume of the bioreactor and stabilizes the cell strain in the required state (‘steady state”), e.g., in the phase of exponential growth [91,96]. Compared to batch cultivation, continuous systems demonstrate a number of important advantages:

- The production of the cell biomass or compound of interest with predetermined and reproducible characteristics due to the stable and thoroughly controlled cultivation conditions;
- The possibility to shift the composition of the cell population and their metabolic activity by manipulating the oxygen supply and nutrient components;
- The possibility to regulate the growth rate of the culture and the concentration of cell biomass within a wide range by changing the flow rate of the nutrient medium.

In addition, the time for equipment preparation is reduced since there is no need for multiple re-sterilizations of tanks.

There are also a number of problems associated with the continuous cultivation [96,97]:

- Difficulties to control the production of secondary metabolites that are not directly correlated with the growth of the cell population;
- Difficulties in providing stable cultivation conditions for cell cultures with high aggregation level and viscosity;
- The risk of losing the culture strain due to cell mutation or due to the auto-selection of cells with a high proliferation rate;
- The high cost and complexity of controlling and automation systems;
- The increased risk of contamination due to long cultivation cycles and the use of additional equipment.

In continuous cultivation, the process is controlled in several ways. The ***chemostat*** is based on measuring and regulating the parameters of the flow entering the system. In this case, the concentration of oxygen or one of the components of the nutrient medium supplied to the bioreactor is fixed at a level at which other nutrient components are in excess. The rate of cell multiplication in the culture is thus limited by a concentration of a regulated component. Wilson et al. [98] and Kurz et al. [99] were among the first to test the chemostat for suspension cultures of *Acer pseudoplatanus* L., *Glycine max* L. Merr., and *Triticum monococcum* L. cells [98,99]. Later, a chemostat was used, to give some examples, for *Petunia hybrida* E.Vilm., *Catharanthus roseus*, and *Nicotiana tabacum* L. cell cultures [100,101,102].

**The turbidostat** is based on measuring the turbidity of the outlet flow. In this case, the change in the optical density of the cell suspension is used to regulate the rate of fresh nutrient medium entering the bioreactor. The turbidostat is rarely used in plant cell culture because of the narrow range of correlation between the optical density of the suspension and the actual cell concentration [15,91,103,104].

The continuous regime is most often used to obtain the growth-associated products of primary and secondary metabolism, to study cell populations in the phase of intensive proliferation, and to culture cell suspensions whose growth is inhibited by cellular metabolic products [15,96,97].

The continuous cultivation regime may be also used to study the metabolic profiles of cell populations in the growth retardation and stationary phases; in this case, cell biomass is not removed from the bioreactor. Such systems are called **closed continuous cultivation systems** [15,91,105]. An important characteristic of the closed continuous mode is the need for a constant supply of nutrient medium and the withdrawal of cell-free culture fluid [91]. In technical terms, this is realized with the help of peristaltic pumps, simple flow rate meters, and tanks for nutrient medium and draining cell-free culture fluid. The technically challenging task here is the continuous separation of cell biomass from the liquid phase, for example, by sedimentation and/or using membranes, while maintaining cell viability and aseptic conditions. Closed continuous systems offer a number of advantages:

- The continuous operation of the system without the problem of cell washout;
- The separated cells are protected from shear stress;
- The possibility of achieving high cell concentrations, up to 30–40 g L^−1^ medium;
- The intercellular contacts are increased in closed cultivation systems.

On the other hand, the closed system for large-scale cultivation of plant cells is limited by a number of negative factors [105]:

- The high chances of cell viability reduction caused by cell separation from the culture fluid or immobilization;
- The difficulties in controlling the growth and biosynthetic parameters of the cell population;
- The significant gradients of nutrients and oxygen within the system in case of cell immobilization or sedimentation;
- The high cost and complexity of the additional equipment.

Cell growth and metabolite production in the closed system are mainly controlled by manipulating the limiting substrate concentration and flow velocity as well as by removing or reducing the concentration of growth-inhibiting metabolites secreted by plant cells to the medium. The varying content of nutrients in the supplied medium also affects the dynamics of intracellular metabolite accumulation. In a closed cultivation regime, cells are not washed out from the system; hence, the flow rate of the nutrient medium may vary within a very wide range, allowing the multifactorial control of the cultured cell population [15,91,96,105]. Closed continuous cultivation systems were used for *Glycyrrhiza inflata* Batalin and *Anchusa officinalis* Thunb. cell culturing [106,107] and to produce recombinant proteins in plant cell suspensions [108].

Many bioreactor systems are hybrids of the batch and continuous cultivation regimes. This includes **periodic substrate-fed cultures** (the periodical addition of the nutrient medium or individual limiting components without the removal of cell biomass) [109,110,111,112], **semi-continuous systems** (the periodic addition of fresh medium, while removing part of the cell suspension) [113,114], **two-stage systems** [70,115], etc. Unlike continuous culture regimes (chemostats in particular), such hybrid systems imply periodic changes in suspension volume and velocity, periodic suspension dilution, as well as varying the specific growth rate, productivity, and other parameters [15,19,91]. Such systems are relatively simple in design and combine the advantages of continuous and periodic cultivation models:

- Multiple options to control and optimize cultivation conditions depending on the phase of the growth cycle, productivity, or culture age;
- Reduced risk of mutations, contamination, or cell washout during cultivation;
- A high degree of substrate utilization;
- The duration of subcultivations may be varied depending on the physiological requirements of the cell population;
- No time-consuming preparation of equipment and inoculum for each new subcultivation cycle.

Combined systems better suit the purpose of upscaling the cultivation process than periodic and continuous regimes. In particular, they are efficient in studies of substrate-limited cell growth, for the cultivation of highly aggregated or slow-growing cell suspensions, and for the production of metabolites whose biosynthesis is not directly associated with growth intensity [19]. For example, a semi-continuous regime was employed by Villarreal et al. [116] for the cultivation of a *Solanum chrysotrichum* C.H. Wright cell suspension in a 10 L airlift bioreactor. The authors observed a 60% increase in biomass and phytochemical productivity in the bioreactor compared to batch cultivation in flasks. Choi et al. [117] compared different regimes for a suspension cell culture of *Thalictrum rugosum* Poir. The highest cell viability, growth rate, and berberine accumulation were observed with semi-continuous cultivation. With suspension cell cultures of *Taxus chinensis* and *Panax notoginseng*, a higher biomass and target metabolite accumulation was observed in the substrate-fed mode compared to periodic cultivation [94,118].

### 3.2. The Use of Different Cultivation Regimes at the Experimental Biotechnological Facility of the IPPRAS

Table 2 presents examples of using different operation regimes for the bioreactor cultivation of suspension cell cultures at the Experimental Biotechnological Facility of the IPPRAS.

**Figure 3:**
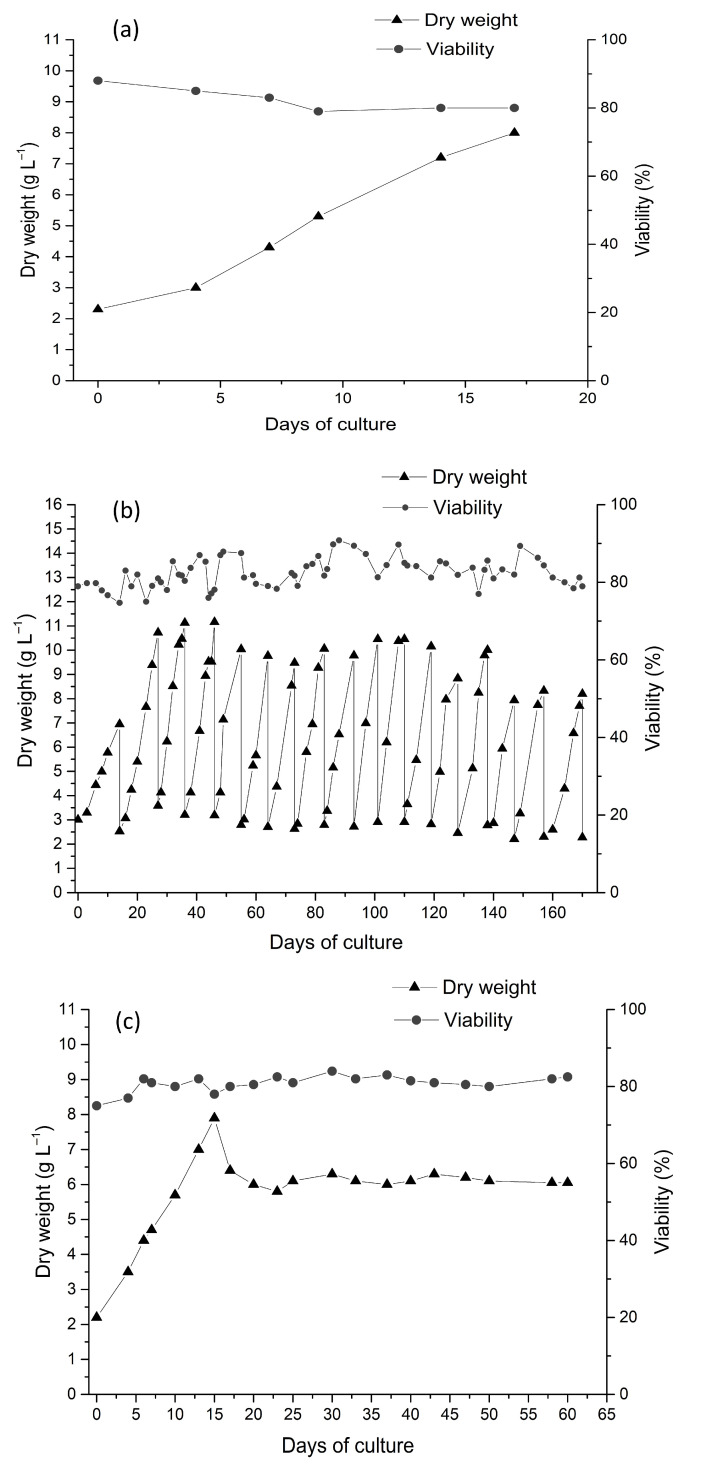
The representative growth curves of plant cell suspensions in bioreactors under different culture regimes: (**a**) *Dioscorea deltoidea*, periodic subculture. The process was finished upon achieving the maximum biomass accumulation on day 17 [121]; (**b**) *D. deltoidea*, semi-continuous regime (growth curve fragment) [121]; (**c**) *Panax japonicus*, continuous cultivation regime (growth curve fragment, medium flow with dilution rate D = 0.11 day^−1^ was initiated on the 15th day of cultivation) [120].

Transferring cell culturing from flasks to laboratory bioreactors is the first step toward upscaling the cultivation process. At the Experimental Biotechnological Facility of the IPPRAS, batch cultivation has been widely used since 1979 in preliminary experiments with laboratory and pilot bioreactors of different types to identify critical factors affecting the productivity of various cell cultures (Figure 3a). The suspension cell cultures tested for batch cultivation were *Dioscorea deltoidea*, *Polyscias filicifolia*, *Stephania glabra* (Roxb.) Miers (synonym of *S. rotunda* Lour.), *Panax japonicus* (T. Nees) C.A.Mey., *Alhagi persarum* Boiss. and Buhse (synonym of *A. pseudalhagi* subsp. persarum (Boiss. and Buhse) Takht., and some others [21,42,43,79,81,83,119]. In these studies, the batch mode was used for the optimization of aeration and agitation regimes, gas mixture compositions, inoculum density, the primary assessment of bioreactor-induced changes in cell aggregation, the growth dynamics, and synthesis of target metabolites under changing cultivation conditions.

Continuous cultivation regimes have been successfully applied for *Dioscorea deltoidea* and *Panax japonicus* cell suspensions (Table 2, Figure 3b). For example, Kandarakov et al. [82] performed a 115-day-long experiment with *Dioscorea deltoidea*, switching between batch and continuous culture regimes and testing four dilution rates. The specific growth rate of cell suspension varied from 0.12 to 0.25 day^−1^ during the exponential growth phase in batch culture and from 0.08 to 0.23 day^−1^ during continuous culture. A viability value of 52–90% was recorded during the whole cultivation cycle. The maximum total furostanol glycoside content was 3.2–4.0%DW. The continuous mode significantly changed the pattern of furostanol glycoside accumulation, likely due to the auto-selection of highly proliferating cells. For the suspension cell culture of *Panax japonicus*, continuous cell cultivation (chemostat) was performed for 70 days with the dilution rate 0.11–0.16 day^−1^. The stable growth of the culture was demonstrated, with the maximum dry cell weight varying depending on the dilution rate within 4.9–7.8 g L^−1^, a viability value of 77–84%, and the total ginsenoside content reaching 5%DW [120].

A closed continuous regime (Table 2) was used by Oreshnikov et al. [85] for a *Dioscorea deltoidea* suspension cell culture. All bioreactors were equipped with peristaltic pumps, vessels for the nutrient medium and cell-free culture medium, and a system to separate the cells from the medium by sedimentation. A maximum dry cell weight of 15.5 g L^−1^ (up to 32 g L^−1^ with an increased medium supply), viability of 60–70%, and maximum total furostanol glycoside content of 4.0–6.0%DW or up to 10%DW depending on the medium concentration were recorded. The dilution rate was maintained at 0.15 day^−1^ (Table 2). Increasing the flow rate to 0.30–0.45 day^−1^ (above the specific growth rate values) led to a reduction in all culture parameters and cell lysis. Moreover, the authors demonstrated that variations in the flow rate, the concentration of nutrient components, and the degree of mechanical stress may be used to purposefully alter and regulate the phases of cell development in the bioreactors.

**Table 2:** The different bioreactor operation regimes for the cultivation of plant cell suspensions used at the Experimental Bitechnological Facility of the IPPRAS.

Species	Bioreactor *	Cultivation Cycle (Days)	Maximum Biomass Accumulation (gDW L^−1^), Cell Viability (%)	Maximum Metabolites Content Achieved	Operating Conditions	Reference
**Periodic (batch) cultivation regime**						
*Dioscorea deltoidea*	Bubble-type bioreactors (no. 1)	21	10.0–11.5 g L^−1^	ND	27 °C, daylight, air flow 0.5–1.0 L min^−1^	[42]
		15–28	9.5–10.0 g L^−1^	ND	27 °C, darkness, air flow 0.4 L min^−1^	[45,79,81]
	MF-107 (no. 5)	21	10.0–11.5 g L^−1^	Diosgenin 7.4–13.7 mg gDW^−1^	27 °C, daylight, stirring rate 350–500 rpm, air flow 0.5–1.0 L min^−1^	[42]
		14–15	9.0–9.5 g L^−1^	Diosgenin 6.2–6.3 mg gDW^−1^	26 °C, darkness, stirring rate 300–500 rpm, pO_2_ 70–90% of saturation volume	[43]
*Polyscias filicifolia*	Bubble-type bioreactors (no. 1)	18	11.0–16.0 g L^−1^	ND	26 °C, darkness, pO_2_–ND	[83]
	MF-107 (no. 5)	24–30	12.8–17.4 g L^−1^	ND	26 °C, darkness, pO_2_–ND	
*Stephania glabra*	Bubble-type bioreactors (no. 2)	21	8.0–16.0 g L^−1^, 75–90%	Stepharin 0.05–0.16%DW	26 °C, darkness, pO_2_ 10–40% of saturation volume	[119]
	75 L tank bioreactor (no. 6)	14	7.0–9.0 g L^−1^, 65–90%	Stepharin, traces	26 °C, darkness, stirring rate 30–65 rpm, pO_2_ 10–40% of saturation volume (single point sparger)	
*Alhagi persarum*	Bubble-type bioreactors (no. 2)	16	13.71 ± 1.84 g L^−1^, 74.1 ± 2.16%	ND	26 °C, darkness, pO_2_ 10–40% of saturation volume	[122]
*Polyscias filicifolia*	75 L tank bioreactor (no. 6)	22	9.3–13.7 g L^−1^, 77–85%	ND	26 °C, darkness, pO_2_ 10–40% of saturation volume (ring-type gas distributor)	[89]
**Continuous cultivation regime**						
*Dioscorea deltoidea*	MF-107 (no. 5)	115	~12.6 g L^−1^, 52–90%	Total furostanol glycosides 3.2–4.0%DW	26 °C, darkness, stirring rate 100–360 rpm, dilution rates (D) 0.14–0.23 day^−1^	[82]
*Panax japonicus* var. *repens*	Bubble-type bioreactors (no. 2)	86	4.9–7.8 g L^−1^, 77–84%	Total ginsenosides 2.5–3.0%DW	26 °C, darkness, pO_2_ 10–40% of saturation volume, D 0.11–0.22 day^−1^	[120]
**Closed continuous cultivation regime**						
*Dioscorea deltoidea*	Fermus-apparatus (no. 3)	57	~14.0 g L^−1^, 60–70%	Total furostanol glycosides 2.0–3.0%DW	26 °C, darkness, pO_2_ 20–60% of saturation volume, stirring rate 20–250 rpm, D 0.15 day^−1^ (days 20–30 and 46–57) **	[85]
	AK-210 (no. 4)	19	15.0–15.5 g L^−1^, 60–80%	Total furostanol glycosides 4.0–6.0%DW	26 °C, darkness, pO_2_ 20–60% of saturation volume, stirring rate 20–250 rpm, D 0.15 day^−1^ (days 7 to 19) **	
		20	30–32 g L^−1^, 62–84%	Total furostanol glycosides 9.5%DW	Same as above, with ×2 medium concentration	[123]
**Semi-continuous cultivation regime**						
*Stephania glabra*	Bubble-type bioreactors (no. 2)	Multicycle 40–60	11.0–16.0 g L^−1^, 78–92%	Stepharin 0.06–0.16%DW	26 °C, darkness, pO_2_ 10–40% of saturation volume ***	[119]
*Dioscorea deltoidea*	Bubble-type bioreactors (no. 2)	Multicycle 182	8.50–12.50 g L^−1^, 80–85%	Total furostanol glycosides 4.2–8.0%DW	26 °C, darkness, pO_2_ 10–40% of saturation volume ***	[124]
	630 L tank bioreactor (no 7)	Multicycle 170	8.87–11.13 g L^−1^, 79–86%	Total furostanol glycosides 7.7–13.9%DW		
*Polyscias filicifolia*	630 L tank bioreactor (no. 7)	Multicycle 112	10.8–16.2 g L^−1^, 79–87%	ND	26 °C, darkness, pO_2_ 10–40% of saturation volume ***	[89]
*Taxus wallichiana*	Bubble-type bioreactors (no. 2)	Multicycle 75	10.5–17.5 g L^−1^, ~90%	Yunnanxane 0.08–0.36 mg gDW^−1^ taxuyunnanine C 0.09–0.34 mg gDW^−1^ paclitaxel 0.06–0.15 mg gDW^−1^	26 °C, darkness, pO_2_ 10–40% of saturation volume ***	[88]
	75 L tank bioreactor (no. 6)	Multicycle 140	9.5–13.0 g L^−1^, ~90%	Synenxane C ~0.55 mg gDW^−1^ yunnanxane ~0.1 mg gDW^−1^		
*Polyscias fruticosa*	Bubble-type bioreactors (no. 2)	Multicycle 204	6.31–7.31 g L^−1^, 70–90%	Ladyginoside A 0.66–0.79 mg gDW^−1^ PFS 0.78–1.03 mg gDW^−1^	26 °C, darkness, pO_2_ 10–40% of saturation volume ***	[86]
*Panax japonicus*	630 L tank bioreactor (no. 7)	Multicycle 115	8.7–10.2 g L^−1^, 86–90%	Total ginsenosides ~7.5%DW	26 °C, darkness, pO_2_ 10–40% of saturation volume ***	[87]

* Numbers in parentheses correspond to the bioreactor numbers in Table 1. ** Equipment to support the closed continuous regime and separate cells from the medium was mounted into the bioreactor. *** To maintain the semi-continuous cultivation, the fresh nutrient medium was fed into bioreactors at the beginning of the stationary growth phase of each subculture cycle until the suspension was diluted to a cell concentration of X0 = ~1.4 gDW L^−1^ for *S. glabra*, X0 = 1.4–2.3 gDW L^−1^ for *P. japonicus*, X0 = 1.5–2.0 gDW L^−1^ for *P. filicifolia* and *P. fruticosa*, X0 = 2.0–2.5 gDW L^−1^ for *D. deltoidea*, and X0 = 2.0–3.0 gDW L^−1^ for *T. wallichiana.* ND—No data; µ—the specific growth rate calculated on a dry weight basis; DW—dry weight; pO_2—_the concentration of dissolved oxygen; X0—initial dry cell biomass weight; D—dilution rate; PFS—28-O-β-D-glucopyranosyl ester of oleanolic acid 3-O-β-D-glucopyranosyl-(1→4)-β-D-glucuronopyranoside. Different strains of *D. deltoidea* cell culture varying in productivity and the ratio of steroidal glycoside content were used in the course of facility operation [39].

The semi-continuous cultivation of plant cell suspensions was successful in bioreactors with a total volume from 10 to 630 L equipped with different stirring devices. Since 1993, repeated experiments have been performed on long-term cell cultivation in all types of bioreactors for different cell suspension cultures. The process of suspension removal and medium refilling was initiated once the suspension reached the cell density corresponding to the beginning of the growth retardation phase. After harvesting a portion of the suspension from a bioreactor, the remaining cell culture was diluted with fresh medium until reaching the minimum cell concentration, allowing further growth without the lag phase (usually above 1.0–2.5 gDW L^−1^). In the course of the cultivation, the physiological parameters and productivity by biomass and target metabolites were evaluated. Stirring and aeration regimes were selected experimentally for each culture considering the following requirements:

- The dissolved oxygen concentration (pO_2_) should remain above 10–15%;
- The stirrer rotation speed should be adjusted to aeration intensity to avoid any “dead” zones in the bioreactor.

The semi-continuous cultivation was successfully developed for the suspension cell cultures of *Stephania glabra*, *Dioscorea deltoidea*, *Polyscias filicifolia*, *Taxus wallichiana*, *Polyscias fruticosa*, and *Panax japonicus* (Table 2, Figure 3c). After the optimization of cultivation conditions, all cell suspensions in bioreactors retained their growth and biosynthetic characteristics at the flask culture level.

## 4. Strategies for Upscaling the Cultivation Process

One of the greatest problems to solve while transferring the process of cultivation from flasks to bioreactors is the optimization of aeration and mixing conditions for each specific cell strain, including the selection of the optimal design of the bioreactor internal space [125,126,127,128].

### 4.1. Mixing

During the cultivation of plant cell suspensions, constant mixing serves two main purposes [19,105]:

- Providing mass transfer between the gas, liquid, and solid phases of the suspension;
- Maintaining homogeneous chemical and physical conditions in the system for a uniform distribution of the nutrients and gases, heat transfer, and dispersion of cell biomass.

In flask cultures, these principles are realized by the constant agitation of cell suspensions on rotary shakers. In bioreactors, mechanical stirring, suspension mixing by compressed air, or a combination of these approaches are applied [105,127]. However, the larger the size and more complex the configuration of the cultivation system, the more difficult it is to achieve uniform mixing. Temperature gradients, fluctuations in the concentrations of limiting substrates, the formation of so-called “dead zones”, etc. are often observed in bioreactors of semi-industrial and industrial scales. The efficiency of mixing is also significantly influenced by the rheological characteristics of the cell suspensions [19,67,127].

Additional difficulties are associated with the sensitivity of plant cells to hydrodynamic and mechanical stress. Hydrodynamic (shear) stress contributes to changes in cell morphology and metabolism, the release of intracellular compounds, and a decrease in viability. For example, a sensitivity to mechanical agitation has been demonstrated for tobacco (*Nicotiana tabacum*) cells cultured in a stirred-tank bioreactor, where the maximum total biomass density decreased by 27% (from 11.8 g to 8.6 gDW L^−1^) with the increasing of the impeller speed from 100 rpm to 325 rpm [129]. Simultaneously, rapid mixing resulted in a high number of visibly damaged and deformed cells [129]. A comparatively higher tolerance to shear stress was shown for *Morinda citrifolia* L. cell culture, where turbine stirring had no detrimental effect on growth. Nevertheless, at a high agitation speed, the accumulation of anthraquinone in the cells was lower than in the flask culture [130]. Hydrodynamic stress led to a decrease in the intracellular adenosine triphosphate (ATP) content and the respiratory activity of *Carthamus tinctorius* L. cell culture, and these changes long preceded cell lysis and membrane damage [131].

The stress effect during stirring is usually minimized by the culture-targeted optimization of bioreactor designs, in particular, by the individual selection of stirrers and gas distribution devices [67,92,127,132,133], the stirring condition (stirrer rotation speed and air supply rate) [67,127,132,133], as well as the selection or creation of cell strains resistant to shear stress, while maintaining high productivity [19,134,135]. For example, with *Coleus blumei* cells, the spiral stirrer provided a 1.5–2 times higher productivity of rosmarinic acid compared to the airlift stirring system and anchor stirrer [35]. Pavlov et al. [136] experimented with the stirrer rotation speed and its effect on the biomass and secondary metabolite productivity of the suspension cell culture of *Lavandula vera* DC, grown in a 3 L bioreactor with a propeller-type stirrer, and they recorded the maximum growth performance at 100 rpm and the maximum yield of rosmarinic acid at 300 rpm. Zhong et al. [137] cultured *Perilla frutescens* (L.) Britton cell suspension in a bioreactor with a marine-type impeller and found that the impeller tip speed of 0.5–0.8 m s^−1^ most favored the accumulation of cell biomass and anthocyanins.

In general, it should be noted once again that the response of plant cell cultures to hydrodynamic stress is individual and depends both on the nature and intensity of the stress and on the physiological state of the cell culture, the age of the population (cells are most susceptible to stress in the lag phase and stationary phase of the growth cycle), the concentration of components in the nutrient medium, the presence of inhibitory metabolites, etc. The hydrodynamic effects of mixing should be particularly considered in large-scale cultivation processes [19,128,138,139].

### 4.2. Aeration

Along with stirring, constant aeration is necessary to achieve the homogeneity of the cell suspension, to increase the rate of the mass exchange of nutrients and products of metabolism, as well as to supply plant cells with oxygen during cultivation [19,68,140,141].

Plant cell respiration is a complex process of thoroughly regulated redox reactions that serve as a source of convertible forms of cellular energy, such as a pH gradient (∆μH^+^) and ATP, and intermediate metabolites involved in various biosynthetic pathways. The electron-transport respiratory chain of plant cells is known to operate two pathways of electron transfer: the main cytochrome pathway (via cyanide-sensitive cytochrome oxidase) and a cyanide-resistant alternative pathway (AP) [142]. Their contribution to the total oxygen uptake varies within a rather wide range and depends on many factors, in particular, on the content and activity of the corresponding enzymes, the degree of electron transfer inhibition, the availability of respiratory substrates, and others [142,143,144,145].

An alternative transport provides an electron transfer from ubiquinone to oxygen, bypassing two parts of the electron-transport chain (complexes III and IV), thus being energetically less efficient than the cytochrome pathway. However, studies confirm the important role of AP in the maintenance of plant cell metabolism [142,143,145]. Moreover, AP regulates the balance of reduced electron transporters, reducing the possibility of reactive oxygen species formation, and it may also promote the active growth of plant cells [146]. The activation of AP in response to negative external factors suggests its participation in signaling mechanisms of plant cell defense against different types of stresses [146,147,148,149].

The measurement of the total oxygen uptake rate is a common method for monitoring the metabolic activity of plant cells during flask or bioreactor cultivation and can adequately indicate the response of cell cultures to changing conditions, including temperature, pH, osmotic stress, nutritional deficiencies, pathogen attack, etc. [143,147,150,151,152]. However, the link between different respiratory metabolic pathways, cell growth, and secondary metabolite biosynthesis in plant cell culture has rarely been studied so far. High cellular respiratory activity promotes intensive growth and biosynthesis processes; therefore, the constant aeration of suspension cultures is necessary to maintain aerobic growing conditions as well as to dissipate possible excess heat generated during the cultivation process [68,145,153].

To prevent an oxygen limitation of cell suspension growth, the dissolved oxygen (dO_2_) concentration in the culture medium is usually maintained at a minimum of 10–15% of saturation [51]. The general O_2_ uptake rate for plant cells has been shown to vary within the range of about 5–10 mmol O_2_ (L·h)^−1^, compared with 10–90 mmol O_2_ (L·h)^−1^ for microbial cells and 0.05–10 mmol O_2_ (L·h)^−1^ or 0.02–0.1×10^−9^ mmol O_2_ (cell·h) ^−1^ for mammalian cells, depending on the individual characteristics of the cell lines, cultivation conditions, phases of the growth cycle, etc. [26,51,94]. For example, for *Thalictrum minus* L. cells actively synthesizing berberine, a twofold increase in the rate of oxygen consumption was observed compared to non-producing cells [154]. Pavlov et al. [136] conducted experiments to study the effects of different concentrations of dissolved oxygen (within 10–50% of the saturation level) on growth and rosmarinic acid production in a cell suspension of *Lavandula vera*. The maximum productivity of both the biomass and rosmarinic acid was observed at dO_2_ 30–50% of the saturation level, while reducing dO_2_ to 10% of the saturation level resulted in a significant decrease in all physiological parameters. This was consistent with the observation of other authors [18,155] that an increased dO_2_ level led to the enhanced respiratory activity and an intensified synthesis of β-glucoronidase and phenolic compounds in *Nicotiana tabacum* cell culture. The effect of dO_2_ on the growth and accumulation of secondary metabolites has also been shown for other cultures, including *Perilla frutescens* and *Catharanthus roseus* [156].

At present, various gas-distributing devices are used for the aeration of the suspension cultures of plant cells grown in bioreactors: single point spargers, rings, lattices, etc. The design of the sparger is selected individually for each cell line/bioreactor type to ensure the optimal air flow without extensive turbulence and promote mass transfer throughout the working volume of the bioreactor. A diameter of the sparger holes of 1–3 mm allows for the efficient dispersion of air bubbles and prevents their clumping and aggregation [127]. Similar to microbiological processes [65,128,157,158], comprehensive studies of aeration efficiency, including automatic measurements of oxygen consumption by cells, and various methods of analyzing the solubility of oxygen and carbon dioxide in the nutrient medium may be useful to evaluate the physiological state of plant cell cultures during cultivation in flasks and bioreactors of different volumes [140,159,160].

### 4.3. Oxygen Mass Transfer Coefficient (K_Lα_)

The difference in the hydrodynamic conditions and mass transfer characteristics of the systems must be taken into account when transferring plant cell cultivation from flasks to industrial bioreactors [19,127,139,161,162]. The upscaling process was previously performed using the “theory of similarities” considering geometric, kinematic, and dynamic properties, each with its own criteria and differential equations describing the cultivation process. However, this approach resulted in an abundant number of criteria that should be satisfied simultaneously during upscaling, which often led to contradictory results; therefore, the principle of geometric similarity was abandoned for simplicity [127,163,164,165].

As mentioned above, the efficiency of bioreactor cultivation is largely determined by the interaction of the growing cell population with the environment, including the transport of nutrient components and gaseous substances from the medium to the cell surface and the removal of cell metabolic products from the cell surface to the medium. This dynamic exchange is, in turn, affected by the hydrodynamic conditions in the bioreactor. Depending on the intensity of the agitation and aeration, the ratio between the turbulent and molecular diffusion changes, causing different mass transfer rates [18,127,163,164,165].

It is crucial to maintain the most suitable conditions of mass transfer, i.e., an optimal hydrodynamic environment, in the process of cell growth in the bioreactor as determined by the conditions of the energy input and the type of bioreactor used [18,127,164,165]. By analogy to microbial and animal cultivation systems, the volumetric coefficient of the oxygen mass transfer *(K_Lα_*) was proposed as one of the key criteria to consider when upscaling the process of plant cell cultivation. For example, the importance of oxygen transfer and its limitations have been demonstrated in scaling up the cultivation of the suspension cell cultures of *Nicotiana tabacum* [129], *Digitalis lanata* [166], *Panax ginseng* [153], and *Taxus chinensis* [167,168]. However, the use of these criteria is only effective when the same macro- and micro-mixing conditions are maintained during the transition from the laboratory to industrial bioreactors.

It is worth noting that the scaling principles for the bioreactor cultivation of plant cells are still being developed. From the technological viewpoint, plant cell cultures are challenging to work with, hence the difficulties to standardize and unambiguous specify the critical scaling parameters for each cell strain [52,54].

### 4.4. Scale-Up Technologies at the Experimental Biotechnological Facility of the IPPRAS

**Figure 4:**
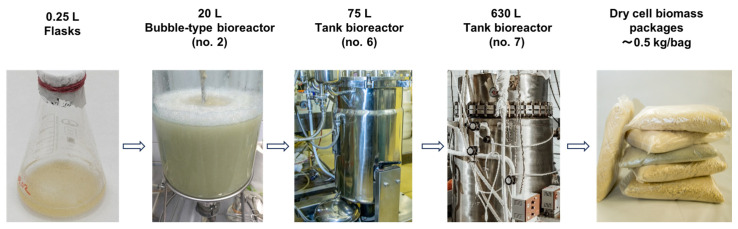
The scheme for upscaling the cultivation of plant cell suspensions in bioreactors at the Experimental Biotechnological Facility of the IPPRAS, from the flask culture to dry biomass product.

As already mentioned, plant cell cultivation in bioreactors is usually focused on scaling up the developed and optimized technological processes from smaller to greater volumes using cell strains with known growth and biosynthetic performance. However, it is usually quite difficult to accurately predict adaptive changes in the cell cultures upon their transfer to bioreactors and to precisely match the geometric and technological aspects of the equipment to the culture’s needs. In our studies, this problem was approached by stepwise analysis of the critical parameters reflecting the physiological state of plant cell cultures at all stages of the upscaling process, from flasks to industrial bioreactors. The development and optimization of the technology for the industrial cultivation of suspension cell cultures was mainly focused on *Dioscorea deltoidea*, *Polyscias filicifolia*, *Panax japonicus*, and *Taxus wallichiana* [21,87,88,89,124,169]. For these cell cultures, changes in growth dynamics and the accumulation of secondary metabolites between the different stages of the upscaling process (Figure 4) were identified and critically analyzed. The changes in the main physiological parameters demonstrated cell strains’ ability to adapt to a new cultivation system. The semi-continuous cultivation was selected for upscaling as the most flexible and productive regime both in terms of biomass and secondary metabolite yield (Table 2). Bubble-type bioreactors (total volume 20–630 L, Figure 4) were chosen for the upscaling scheme based on the highest growth and biosynthetic characteristics of the cell strains observed in this type of bioreactors [83,87,88,124]. Bioreactors with a 20 L volume were inoculated directly from flasks. The cell suspension produced in bioreactors of the smaller volume was used to inoculate the larger ones (Figure 4). Our results demonstrated that ring-type aerators were more suitable than single point spargers for maintaining optimal mass transfer conditions in bioreactors of different volumes. Cultivation in mechanically stirred bioreactors usually resulted in lower growth and biosynthetic characteristics and could only be recommended for short-term use.

Important information about the physiological state of the cell population during the cultivation and upscaling process can be obtained by analyzing cells’ respiration activity. In particular, a correlation between the changes in the respiration intensity and the dynamics of secondary metabolite accumulation was observed for *Dioscorea deltoidea* and *Panax japonicus* cell cultures: the maximum rate of oxygen uptake was recorded before the beginning of active metabolite synthesis, i.e., in the lag phase for *D. deltoidea* and in the exponential phase for *P. japonicus*. In other words, during plant cell cultivation, the oxygen supply rate should be set depending on the culture’s biosynthetic activity [124,170]. Moreover, the activity of alternative oxidase in *Dioscorea deltoidea* cell culture was significantly affected by the cultivation conditions and, in particular, by the mass-exchange characteristics of the bioreactors. When the cell suspension was cultured in bubble-type bioreactors of different volumes with a different sparger configuration, the highest level of cyanide-resistant respiration was recorded for the 20 L bioreactors with a single point aerator, which corresponded to minimum *K_Lα_* values and the lowest production of cell biomass and furostanol glycosides. Probably, this effect was due to a non-uniform distribution of oxygen in the culture medium [124].

The reproducibility of the main growth and biosynthetic characteristics of selected strains during prolonged cultivation in bioreactors is fundamentally important for the development of industrial technologies. The scale-up cultivation of the suspension cell cultures of *Dioscorea deltoidea*, *Polyscias filicifolia*, and *Panax japonicus* using the semi-continuous regime has been repeated multiple times during the past 20 years, with similarly high growth and biosynthetic parameters successfully reproduced for all of them (Table 3).

The cell biomass of *Dioscorea deltoidea* and *Panax japonicus* produced in industrial bioreactors contained essential macro- (K, Ca, Mg, Na) and micro- (Zn, Mn, Fe, B, Al, Cu) elements in dietary safe concentrations [21,87]. Toxicology analysis on in vivo models revealed little or no effect of the cell biomass of these cultures on the animal state, organ weights, and the hematological and biochemical parameters of the blood [21,87]. Phytopreparations based on the cell culture extracts of bioreactor-produced *P. japonicus*, *D. deltoidea*, and *Tribulus terrestris* L. also demonstrated positive effects in rats with induced type 2 diabetes mellitus and diet-induced obesity [171,172].

## 5. Conclusions

The Experimental Biotechnological Facility of the IPPRAS was established in the 1970s as the first Russian center for biotechnological research and the production of plant cells and phytochemicals. After multi-year tests and the adaptation of bioreactors with different designs and operation regimes, a cascade of bioreactor pipelines from the laboratory (20 L) to industrial (630 L) scale were developed and optimized for cell cultures of medicinal plants, including *Polyscias filicifolia*, *Panax japonicus*, *Dioscorea deltoidea*, and *Taxus wallichiana*. The main growth and biosynthetic characteristics for all tested cell strains remained stable and were successfully reproduced during repeated long-term bioreactor cultivation. The maximum duration of semi-continuous cultivation in the industrial bioreactor reached 170 days. During the scaling up of the cultivation process to industrial volumes, all strains maintained an active synthesis of target metabolites (ginsenosides, furostanol-type glycosides, triterpene glycosides of the oleanane type, and taxanes) at a sufficiently high level, mostly corresponding to those recorded for flask cultures [21,87,171,172]. A minor decrease in the productivity of secondary metabolites and a reduction in all physiological parameters was observed only for the pilot (75 L) bioreactor with a single point sparger combined with mechanical agitation, which was likely due to cell culture response to intense shear stress. The highest productivity of secondary metabolites was recorded for the industrial bubble-type bioreactor with a ring sparger, and the hydrodynamic conditions of this model were considered the most appropriate for the cultivation of plant cell suspensions.

In general, the scale-up experiments have demonstrated the high sensitivity of plant cell suspension cultures to even minor modifications in cultivation systems. The level of biomass and secondary metabolite accumulation was notably affected by bioreactors’ technical characteristics, such as the design, aeration, and mixing intensity, as well as their cultivation conditions (media composition, inoculum, etc.). This is consistent with the literature data [52,54,56,164,176,177,178] on the lack of uniform and comprehensive scaling criteria for geometrically and structurally dissimilar bioreactor systems. Much of the success depended on the ability of the cells to adapt to the stress caused by bioreactor cultivation. Modifications of the cultivation conditions had a stronger effect on the biosynthetic characteristics of the cell cultures than on the growth parameters. It was critically important to tailor both the operation regime and the cultivation conditions, particularly the aeration and mixing rates, depending on the bioreactor type and cell strain.

In conclusion, the research team of the Experimental Biotechnological Facility of the IPPRAS developed effective systems, methods, and criteria for scaling up the plant cell cultivation process from flasks to bioreactors of industrial volumes. These results will be helpful for the development of green biotechnological platforms and production, assessments, and the certification of plant cell biomass as a sustainable component of functional foods, food additives, and natural health products.

## Figures and Tables

**Table 1:** Bioreactors of the Experimental Biotechnological Facility of the IPPRAS.

No.	Bioreactor	Material	Total/Working Volume, L	Mixing	Sparger Type	Impeller Type	Manufacturer	Advantages (A), Disadvantages (D)	Exploitation Period
**Laboratory (bench-top) bioreactors**									
1.	Bubble-type bioreactors	Glass	1.5/1.0, 3.0/2.5	Aeration	Single point sparger, ∅~2 mm *	n/a	IPPRAS, Moscow, Russia	A: Easy upscaling, simple construction, low cost D: Small volume, intense foaming, non-optimal mass transfer, application is limited to fine-aggregated, non-foaming cell lines	Until 2014 [84]
2.		Glass	10/7, 20/15		Single point sparger, ∅~6 mm				Currently in use [86]
3.	Fermus-apparatus	Glass + stainless steel	8/6	Magnetic stirrer + aeration	Single point sparger, ∅~4 mm	Open turbine impeller	R&D Center “Bioavtomatika”, N. Novgorod, Russia	A: Highly efficient mass transfer D: Intense shear stress, foaming, non-optimal magnetic drive configuration, limited options for modification, higher chances for contamination due to construction specifics	Until 1995 [85]
4.	AK-210	Glass + stainless steel	10/8	Magnetic stirrer + aeration	Single point sparger, ∅~4 mm	Open turbine impeller	R&D Bureau, Pushchino, Russia		Until 1995 [85]
5.	MF-107	Glass + stainless steel	7/5	Magnetic stirrer + aeration	Single point sparger, ∅~4 mm	Three-impeller stirrer (two open turbine impellers and one marine-type impeller)	New Brunswick, USA		Until 2000 [83]
**Pilot-scale bioreactors**									
6.	Tank bioreactor	Stainless steel	75/50	Magnetic stirrer for media sterilization, aeration for cell cultivation	Single point sparger, ∅~6 mm or ring-type gas distributor ∅~200 mm with multiple holes ∅~1 mm	Marine-type impeller	Electrolux, Sweden	A: Highly efficient mass transfer, suitable for viscous cell suspensions D: Intense shear stress, high energy cost due to mechanical agitation	Currently in use [87,88,89]
**Industrial-scale bioreactors**									
7.	Tank bioreactor	Stainless steel	630/550	Magnetic stirrer for media sterilization, aeration for cell cultivation	Ring-type gas distributor ∅~750 mm with multiple holes ∅~1 mm	Marine-type impeller	1T series, “EBEE” Research & Manufacturing facility, Yoshkar-Ola, Russia	A: Highly efficient mass transfer, suitable for viscous cell suspensions D: Intense shear stress, high energy cost due to mechanical agitation	Currently in use [21,87,89]

* here and further in the table, the inner diameter is specified.

**Table 3:** Examples of cell culture strains adapted for large-scale (630 L) bioreactor cultivation at the Experimental Biotechnological Facility of the IPPRAS to produce useful health products *.

Suspension Cell Culture	Metabolites Produced	Biological and Pharmacological Activities	Reference
*Dioscorea deltoidea*, strain DM-05-03	25(S)- and 25(R)-deltoside isomers, 25(S)- and 25(R)-protodioscin isomers, dioscin	Bioreactor-produced cell biomass was assessed for elemental composition and toxicology, and it demonstrated positive effects in rats with induced type 2 diabetes mellitus and obesity	[21,124,171,172]
*Panax japonicus*, strain 62	Ginsenosides: PPD: Rb1, Rc, Rb2/Rb3, Rd; PPT: Rg1, Re, Rf; OA: R0, chikusetsusaponin IVa; malonylated derivatives of ginsenosides	Bioreactor-produced cell biomass was assessed for elemental composition and toxicology and exhibited hypoglycemic and hypocholesterolemic activity in rats with diet-induced obesity	[87,169,173]
*Polyscias filicifolia*, strain BFT-01-95	Triterpene glycosides of the oleanane type: PFS, ladyginoside A, polysciosides A–E	Bioreactor-produced cell biomass has documented adaptogenic and anti-teratogenic activities and is currently used in commercial food supplements	[89,174,175]

*—upscaling scheme as in Figure 4. PPD—20(S)-protopanaxadiol group; PPT—20(S)-protopanaxatriol group; OA—oleanolic acid group; PFS—3-O-[β-D-glucopyranosyl-(1→4)-β-D-glucuronopyranosyl] oleanolic acid 28-O-β-D-glucopyranosyl ester.

## Data Availability

No new data were generated during this research.
